# Exploring Nucleic Acid Nanozymes: A New Frontier in Biosensor Development

**DOI:** 10.3390/bios15030142

**Published:** 2025-02-24

**Authors:** Keren Chen, Zaihui Du, Yangzi Zhang, Ruobin Bai, Longjiao Zhu, Wentao Xu

**Affiliations:** Food Laboratory of Zhongyuan, Key Laboratory of Precision Nutrition and Food Quality, Department of Nutrition and Health, China Agricultural University, Beijing 100193, China; yvonnechenkr@cau.edu.cn (K.C.); duzaihui@cau.edu.cn (Z.D.); yz.zhang@cau.edu.cn (Y.Z.); bairuobin@zyfoodlab.com (R.B.)

**Keywords:** nucleic acids, nanozymes, aptamers, biosensors, enzyme-like properties

## Abstract

With the growing interest in nucleic acids and nanozymes, nucleic acid nanozymes (NANs) have emerged as a promising alternative to traditional enzyme catalysts, combining the advantages of nucleic acids and nanomaterials, and are widely applied in the field of biosensing. This review provides a comprehensive overview of recent studies on NAN-based biosensors. It classifies NANs based on six distinct enzymatic activities: peroxidase-like, oxidase-like, catalase-like, superoxide dismutase-like, laccase-like, and glucose oxidase-like. This review emphasizes how the catalytic activity of nanozymes is significantly influenced by the properties of nucleic acids and explores the regulatory mechanisms governing the catalytic activity of NANs. Additionally, it systematically reviews important research progress on NANs in colorimetric, fluorescent, electrochemical, SERS, and chemiluminescent sensors, offering insights into the development of the NAN field and biosensor applications.

## 1. Introduction

A biosensor is an analytical device that combines a biological recognition element (such as an enzyme, antibody, or nucleic acid) with a physicochemical detection system for the detection and quantification of a specific analyte [1]. The biological recognition element specifically interacts with the target analyte, while the detection system converts this interaction into a measurable signal. Biosensors are often considered for portable on-site detection and point-of-care diagnosis due to their advantages of simple operation, fast and accurate response, cost-effectiveness, and ease of miniaturization. Biosensors are now considered to be a reliable alternative to standard laboratory testing methods and have been widely used in areas such as food, environment, and healthcare [2,3,4]. Current innovations in biosensor design focus on enhancing sensing characteristics, reducing sample volume, and minimizing user intervention.

Nanozymes represent a class of nanomaterials with intrinsic catalytic properties similar to natural enzymes. Their unique properties make them promising candidates for applications in biosensors, particularly for use in performance improvement [5,6]. Various nanozymes, including those based on noble metals, transition metals, carbon, and metal–organic frameworks, have been discovered to possess peroxidase (POD)-like or/and oxidase (OXD)-like activities, enabling their use in the direct detection of H_2_O_2_, glucose, ascorbic acid (AA), and other analytes [7]. Although nanozymes have advantages over natural enzymes in biosensor applications, such as pH and temperature tolerance and low cost, the green synthesis, enzyme activity regulation, and catalytic specificity of nanozymes still need further study.

Nucleic acid nanozymes (NANs) are a novel class of nanomaterials that combine the specificity of nucleic acids with catalytic properties similar to natural enzymes, making them promising candidates for biosensor applications. Their high stability, ease of functionalization, and resistance to environmental factors such as pH and temperature give them an advantage over traditional enzymes in biosensing platforms [8]. Additionally, their synthetic nature allows for flexible modification and optimization, offering enhanced selectivity and sensitivity [9].

**Figure 1:**
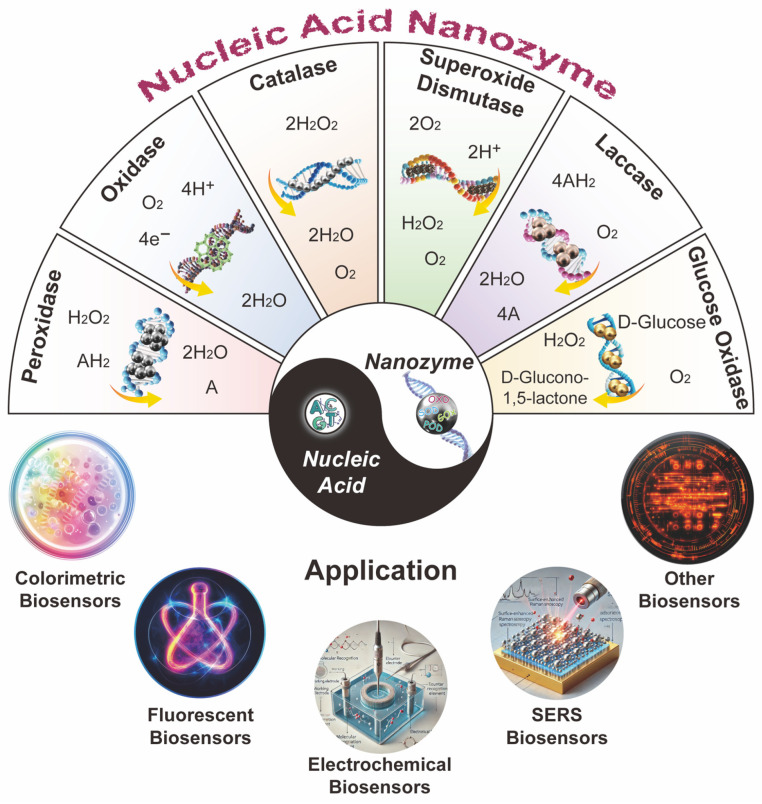
Taiji schematics of NANs in different enzyme activity types and their applications in biosensors.

This review primarily focuses on the advancements in biosensor technology, with a particular emphasis on biosensors based on NANs (Figure 1). It begins by exploring the advantages of NANs in enhancing biosensor performance, followed by a comprehensive classification of NANs based on their six distinct enzymatic activities. The review also provides a detailed analysis of how nucleic acids influence the catalytic activity of nanozymes and offers an in-depth exploration of the regulatory mechanisms governing their properties. Additionally, it extensively reviews the applications of NANs in five types of biosensors, providing profound insights into future directions for optimizing NAN-based biosensors for practical, real-world use. This work aims to serve as a valuable reference for the design and application of nanozyme-based biosensors.

## 2. Advantages of NANs

Nucleic acids, essential biomolecules, are composed of nucleotides, which consist of a pentose sugar, a phosphate group, and a nitrogenous base [10]. The unique structural and physicochemical properties of nucleic acids enable them to interact with inorganic nanomaterials via various forces, including coordination, π-π stacking, hydrophobic interactions, and electrostatic forces [11,12]. The effects of nucleic acids on nanozymes are complex, and the literature contains conflicting reports. Some studies have shown that single-stranded DNA (ssDNA) can enhance the POD-like activity of Fe_3_O_4_ and CeO_2_ nanoparticles, while other studies have found that ssDNA can inhibit POD-like activity [13,14,15]. In order to better understand the effects of nucleic acids on nanozymes, it is also necessary to consider the type of DNA, buffer conditions, and reaction system. A clear understanding of the various intrinsic and extrinsic factors that regulate the enzymatic activity of nanozymes is a prerequisite for the precise design of NAN biosensors.

Although the regulatory mechanism of nucleic acids on nanozymes remains to be further elucidated, the advantages of NANs can be summarized as follows due to the physicochemical properties of nucleic acids that can endow nanozymes with new functions:

(1) The involvement of nucleic acids makes the synthesis of nanozymes simple, versatile, and precisely controllable. Nucleic acids are naturally occurring nanoscale materials whose dimensions depend on the number of base pairs [16]. DNA has a high affinity for cationic metals due to the Lewis acid–base interactions between the nucleic acid bases and the metal ions. This property allows DNA to act as a template and efficiently assemble metal ions, thereby forming stable metal nanozymes [3]. The flexibility of DNA is modulated by the specific sequence and stacking interactions of its constituent base pairs. DNA origami technology enables precise customization and assembly of nucleic acid spatial structures, which can be used to precisely control the enzymatic activity of nanozymes [17,18].

(2) The negatively charged phosphate backbone can effectively enhance the activity and stability of nanozymes [19]. The negatively charged phosphate backbone of nucleic acids stabilizes nanozymes by preventing particle aggregation, enhancing catalytic efficiency through improved electron transfer, and increasing thermal and chemical stability in challenging environments. It also serves as a scaffold for precise catalytic site arrangement, further boosting enzymatic activity.

(3) The rich chemical functionalities in nucleic acids can endow nanozymes with more functionalities, including providing binding sites for analytes and functionalization through modification with various moieties on their surfaces [20,21]. The abundant functional groups in nucleic acids can serve as primers for chemical branching, changing the spatial structure and physical properties [22].

(4) The good biocompatibility, biodegradability, and non-cytotoxicity of nucleic acids can improve the biosafety of nanozymes. Nucleic acid molecules are naturally present in living organisms and can be easily recognized by the body [12]. Additionally, nucleases that can hydrolyze nucleic acids are present, making them relatively safe. Moreover, aptamer-templated nanozymes have been reported to exhibit targeting ability and sustained-release properties, reducing the toxicity of the nanomaterials and ensuring biosafety [23,24].

(5) Nucleic acids have the ability to undergo exponential amplification in vitro, which enables their large-scale production to meet the demands of green synthesis of nanozymes and cost reduction [22,25]. Nucleic acids can be exponentially amplified through techniques like polymerase chain reaction (PCR), rolling circle amplification (RCA), and strand displacement amplification (SDA), enabling rapid, cost-effective, and large-scale production of uniform templates for nanozyme synthesis. This scalability supports industrial applications, ensures customization, and promotes sustainable, eco-friendly manufacturing for fields like biomedicine and environmental monitoring.

In summary, capitalizing on their exceptional tunable catalytic performance, stability, scalability, biocompatibility, and cost-effectiveness, nanomaterials with enzyme-like properties have found widespread applications in diverse fields, spanning from food safety to environmental monitoring and biomedicine.

## 3. Classification Based on Enzyme-like Activity

According to the nature of enzymatic reactions, the International Union of Biochemistry and Molecular Biology (IUBMB) classifies enzymes into six categories: oxidoreductases, transferases, hydrolases, lyases, isomerases, and ligases. With the continuous advancement of nanozyme research, nanozymes have been found to possess various enzyme-like functions, including oxidoreductase-, hydrolase-, and ligase-like activities. Among these, oxidoreductase-like activities are the most common, particularly POD-, OXD-, catalase (CAT)-, superoxide dismutase (SOD)-, laccase- and glucose oxidase (GOx)-like activities. This paper mainly focuses on the enzyme-like properties of NANs.

### 3.1. POD-like

POD-like are a class of oxidoreductases that utilize hydrogen peroxide (H_2_O_2_) as an electron acceptor to catalyze the oxidation of substrates. H_2_O_2_ activation by POD-like occurs via two catalytic mechanisms: the Fenton-type and the Poulos−Kraut mechanisms [26,27]. The POD properties are generally believed to require the production of reactive oxygen species (ROS) (e.g., •OH, and O_2_^•−^) to oxidize substrates. Currently, the largest number of nanozymes with POD-like activity have been reported, and new nanozymes are constantly being discovered.

The initial discussion will focus on the role of aptamers in modulating the POD-like activity of nanozymes. Nucleic acid aptamers can adsorb onto nanomaterials through various interactions, including electrostatic interactions, hydrogen bonding, and van der Waals forces [28,29,30]. When the aptamer is bound to its target molecule, it undergoes a conformational change that reduces its affinity for the nanomaterial. By controlling the binding affinity between the aptamer and the nanomaterial, it is possible to achieve efficient control of nano-enzyme activity. For example, when DNA is combined with MoS_2_ nanosheets (MoS_2_ NSs), the affinity for the substrate TMB is increased, which further accelerates the electron transfer from TMB to H_2_O_2_. Therefore, the POD-like activity of MoS_2_ NSs is significantly enhanced, which is 4.3 times higher than that of bare MoS_2_ NSs [31]. Fan et al. achieved enhanced POD-like activity of GO/Au using a PBP2a aptamer, which they attributed to aromatic stacking and electrostatic interactions with the substrate TMB [32]. The enzyme activity of the GO-CTAB-AuNP hybrid nanozyme was also enhanced using amphetamine-type stimulants’ specific aptamers [33]. The three-dimensional (3D) branched carbon nitride nanoneedle (3DBC-C_3_N_4_) also provides interfaces for the reversible conjugation of OTC aptamers, enhancing their colorimetric sensitivity [34].

**Figure 2:**
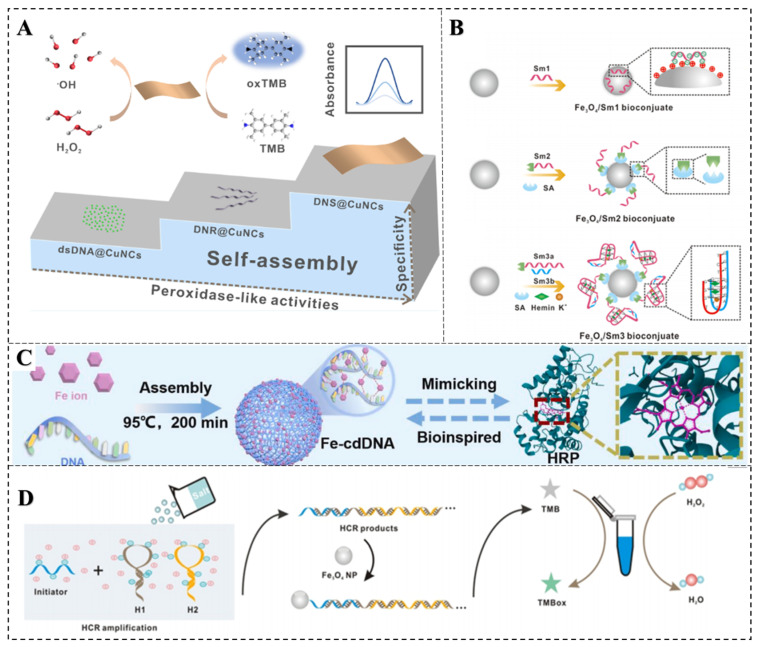
Typical nanomaterials with POD-like activities. (**A**) DNS@CuNCs [18]; (**B**) Fe_3_O_4_-Sm1/2/3 [37]; (**C**) Fe-cdDNA [39]; (**D**) HCR/Fe_3_O_4_ [41].

Subsequently, we discussed the influence of nucleic acids on the specificity of nanozymes with POD-like activity. Compared to natural enzymes, nanozymes have inherent limitations in specificity and activity. To improve the specificity of nanozymes, Li et al. employed a DNA framework-templated self-assembly strategy to address the POD-like specificity issue (Figure 2A) [18]. The synthesized DNA nanoribbon-templated CuNCs (DNR@CuNCs) and DNA nanosheet-templated CuNCs (DNS@CuNCs) exhibited exceptional catalytic activity compared to dsDNA-templated CuNCs (dsDNA@CuNCs). DNS@CuNCs showed the highest enzymatic activity, approximately 2-fold higher than DNR@CuNCs and 12-fold higher than dsDNA@CuNCs. Furthermore, DNS@CuNCs displayed the highest POD-like specific activity, reaching 1.79 × 10^3^ U mg^−1^ among the three. These differences in catalytic activity and specificity were attributed to the content of intermediates generated after the introduction of nanozymes. Moreover, while Ti_3_C_2_, a typical MXene, possesses excellent hydrophilicity, electrical conductivity, functionalization capability, and biocompatibility, it lacks specific recognition functionality and has relatively low intrinsic POD-like activity. The oligonucleotide adsorption can greatly improve the catalytic activity of Ti_3_C_2_ [35]. Furthermore, the integration of abundant unsaturated Ti center edges and residual Mn^2+^ within the single-layer porous Ti_3_C_2_ framework through microwave combustion technology enhances the adsorption capacity of the DNA aptamer, thereby improving the catalytic efficiency of the Ti_3_C_2_ nanoparticles [36].

Additionally, key factors in controlling the catalytic activity of nanozymes also include the coverage density, salt concentration, and curvature present on the engineering biological interfaces [13]. As an example, engineering nanointerfaces with ssDNA was found to enhance the POD-like characteristics of Fe_3_O_4_ nanoparticles in the oxidation of TMB [37]. Moreover, the catalytic activity of Fe_3_O_4_ NPs could be precisely adjusted by modifying the inorganic surface with DNA ligands of varying structures, including affinity coupling methods and the physical spacer of DNA (Figure 2B) [38].

In addition, nucleic acids can serve as templates, directly guiding the synthesis of nanozymes. In this study, a coordination-driven self-assembly method is developed to synthesize Fe-cdDNA nanozyme by mixing DNA aqueous solution and Fe(II) (Figure 2C) [39]. The abundant phosphate binding sites and the presence of N and O atoms within nucleobases facilitate the coordination interaction between DNA and Fe(II). The obtained nanozymes exhibit adjustable POD-like catalytic properties and excellent chemical stability. By controlling the metal ions, DNA concentration, and DNA length, the morphology and catalytic activity of Fe-cdDNA can be predictably and systematically regulated. The catalytic mechanism is proposed wherein H_2_O_2_ is initially adsorbed on Fe-cdDNA and then activated by Fe-cdDNA to generate O_2_^•−^, which rapidly transfers electrons to 3,3′,5,5′-tetramethylbenzidine (TMB), completing the oxidation of TMB to oxidized TMB (oxTMB). Moreover, research indicates that DNA is also instrumental in improving the enzymatic activity of Fe-N-C single-atom nanozymes [40]. The DNA-assisted enhancement is attributed to the electrostatic attraction between the Fe-N-C nanozyme and the substrate, leading to increased substrate affinity.

While ssDNA is commonly used to functionalize nanozymes, studies have also investigated the application of more rigid DNA structures, such as DNA duplexes, HCR products, PCR products, and DNAzyme. Zeng et al. demonstrated that DNA could serve as a versatile and powerful tool for the precise regulation of catalytic nanomaterials, where the HCR product-treated Fe_3_O_4_ NPs and gold nanoparticles (AuNPs) exhibited greatly enhanced enzymatic activities toward TMB (Figure 2D) [41]. Normally, the conjugation of long biomacromolecules induces the aggregation of individual nanozyme particles, leading to the loss of their POD-like activities. For example, human genomic DNA (hgDNA) can irreversibly aggregate Fe3O4 nanoparticles and cause the loss of their activity. However, after the Fe_3_O_4_ was immobilized on silica microspheres to synthesize anti-aggregation nanozymes (Fe_3_O_4_ NP@pSiO_2_), not only did the hgDNA not aggregate the nanozymes, but it also enhanced the enzymatic activity of Fe_3_O_4_ NP@pSiO_2_ according to the hgDNA concentration [42]. Moreover, encapsulating AuNPs with a DNA corona leads to the creation of a new type of artificial enzyme—the “coronazyme”, which merges the properties of both nanozymes and DNAzymes. These coronazymes demonstrate enhanced catalytic efficiency and good specificity [43].

### 3.2. OXD-like

The catalytic mechanism of OXD-like, which directly catalyzes the generation of •OH from O_2_, has received extensive attention due to the avoidance of unstable and toxic H_2_O_2_ substrate. Typical examples include CeO_2_ [15], Fe-N-C [44], NiO [45], RuNPs [46], etc.

**Figure 3:**
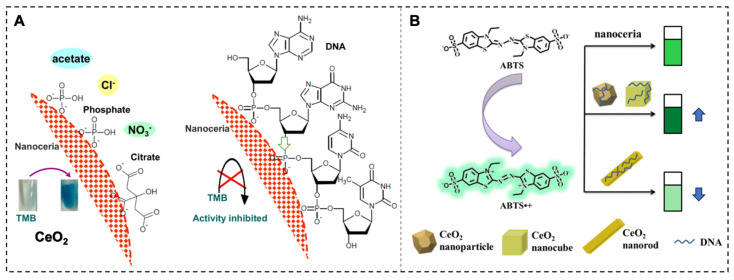
CeO_2_ OXD-like activity and its regulation. (**A**) DNA adsorption inhibits the OXD activity of nanoceria by blocking substrate accessibility [14]. (**B**) DNA enhances the OXD-like activity of CeO_2_ nanoparticles and nanocubes but reduces it in nanorods [48].

Cerium oxide nanoparticles (CeO_2_ nanoparticles) possess mixed valence states of Ce^3+^ and Ce^4+^, which endow them with unique redox properties [15,47]. Research suggests that the redox state of CeO_2_ nanoparticles has been shown to be influenced by phosphate, and several phosphate-containing molecules have been reported to modulate its OXD-like activity. Studies have reported that nucleoside triphosphates can enhance the OXD-like activity of nanoceria and that this enhancement effect is dependent on the type of NTP. DNA with phosphate groups can be effectively adsorbed on CeO_2_ regardless of its base composition, but longer DNA adsorbs more tightly. Fluorescence quenching reflects that ssDNA adsorbs more effectively than dsDNA. DNA adsorption is not only realized by electrostatic interaction but also through the combination of its phosphate backbone with cerium via Lewis acid–base interaction. DNA adsorption blocks the surface accessibility of substrate molecules, thus inhibiting the OXD activity of nanoceria (Figure 3A) [14]. However, with the deepening of research, the morphology of the nanoceria plays a crucial role in determining its OXD-like activity. Different morphologies of nanoceria have different surface structures and properties, which can affect their interaction with DNA and, consequently, their OXD-like activity. DNA can enhance the OXD-like activity of CeO_2_ nanoparticles and nanocubes but reduce the OXD-like activity of nanorods (Figure 3B) [48]. Aptamer sequences with target recognition capabilities can also interact with the surface of CeO_2_, thereby modulating its enzymatic activity. Gonca et al. designed a redox activity switch for CeO_2_ based on an OTA aptamer, which was used for the detection and quantification of a target biomolecule [49]. Moreover, some reports have demonstrated the inhibitory effect of double-stranded PCR amplicons on the OXD activity of CeO_2_, which has been successfully applied in colorimetric and electrochemical biosensors based on this property [50,51]. These reports show that the effect of nucleic acid sequences on the OXD activity of CeO_2_ seems to be inconsistent, which is mainly due to the fact that the activity of CeO_2_ is affected by multiple factors (such as the buffer type, salt concentration, pH, etc.). Therefore, when utilizing their enzymatic activity, we need to pay special attention to the fine control of the reaction conditions.

### 3.3. Catalase-like

A class of nanomaterials known as CAT-like nanozymes possess inherent catalase activity, enabling them to catalyze the decomposition of H_2_O_2_ into molecular water and oxygen. Their activity can be readily modulated by manipulating their morphology, size, defects, and other properties (e.g., pH and temperature) [52,53]. CAT-like nanozymes have attracted considerable attention in recent years due to their potential as effective substitutes for natural enzymes in a wide range of applications, particularly in biosensing [54,55,56]. In 2009, gold-platinum bimetallic nanoparticles (CP-Au/Pt) stabilized by pectin were the first nanozymes demonstrated to possess CAT-like activity [57].

**Figure 4:**
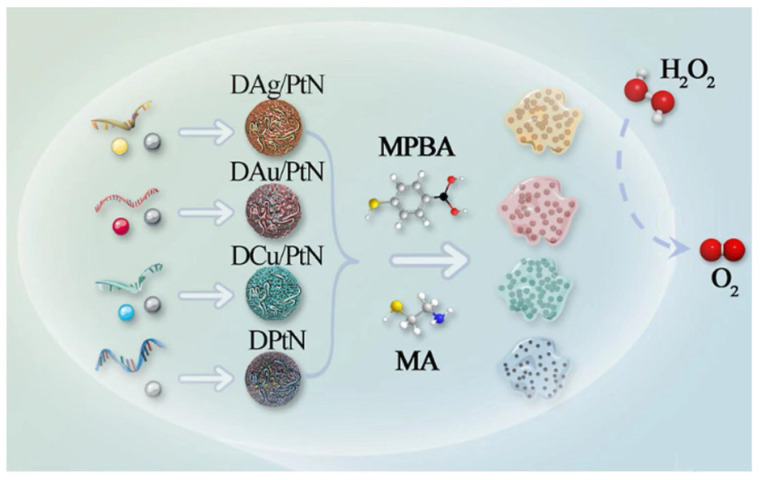
Functionalized DNA-nanozymes with CAT-like [58].

Jiao et al. utilized four DNA nanozymes (DAg/PtN, DAu/PtN, DCu/PtN, and DPtN) functionalized with 4-mercaptophenylboronic acid (MPBA) and β-mercaptoethylamine (MEA) to achieve CAT-like activity (Figure 4). The boronic acid groups of MPBA can strongly bind to cis-diol molecules and phosphate of peptidoglycan in Gram-positive bacteria to form borate esters (pH ≥ 7.0), while MEA can strongly interact with negatively charged groups on the bacterial surface through electrostatic interactions. Based on this, the four functionalized DNA nanozymes can interact with nine bacteria (four Gram-negative and five Gram-positive) to different extents through non-specific interactions, which affects their ability to catalyze H_2_O_2_ to produce O_2_, thereby changing the pressure inside the sealed tube to generate unique “fingerprints”. Therefore, a pressure sensor array was successfully constructed to support the portable multiplex detection of foodborne pathogens [58].

### 3.4. SOD-like

Metalloenzymes known as SOD catalyze the dismutation of superoxide radicals (O_2_^•−^) into H_2_O_2_ and oxygen, serving as the primary defense against ROS-mediated damage. SODs thus hold promise for treating oxidative stress-related diseases [59]. Currently reported nanozymes with SOD-like activity include CeO_2_ [60]; fullerenes [61]; Cu-SAzyme [62]; and various metal-containing oxides, carbides, nitrides, and sulfides of Fe [63], Au [64], Pt [65], etc. The main mechanisms by which various nanozymes generate SOD-like activity include the protonation of O_2_^•−^ and the adsorption and rearrangement of HO_2_• on the nanozyme surface. O_2_^•−^ can easily capture protons in water to form HO_2_• and OH^−^, and the adsorption of HO_2_• on the nanozyme surface is likely to be a strongly exothermic process. In other words, once HO_2_• is adsorbed on the surface, it can easily be converted to O_2_* and H_2_O_2_*. Subsequently, O_2_* and H_2_O_2_* are converted back to O_2_ and H_2_O_2_ [66].

Aptamer-modified atomically precise gold Au_25_ nanoclusters (Apt-Au_25_ NCs) exhibit ultrahigh SOD-like and CAT-like activities, good targeting ability, and low cytotoxicity. Moreover, they possess good thermal and pH stability compared with natural SOD and CAT. They have been successfully applied to scavenge ROS in white adipocytes, showing great potential for the treatment of obesity and related diseases [67].

### 3.5. Laccase-like

Laccase catalyzes the oxidation of various substrates through a four-electron transfer involving four copper ions in its catalytic center [68,69]. The majority of research on laccase-like nanozymes has centered on “copper” as the crucial element, as copper is indispensable for the active site of laccase [70]. Tannic acid coordination copper (Cu-TA), platinum nanoparticles (PtNPs), and manganese oxide (Mn_3_O_4_) are other reported nanomaterials that exhibit laccase-like activity. Most of the reported laccase mimics, owing to their excellent oxidation performances, have been mainly applied for the analysis of phenolic compounds.

**Figure 5:**
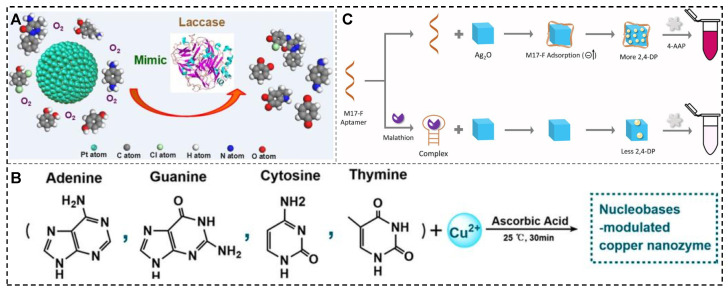
Typical nanomaterials with laccase-like activities. (**A**) Oligonucleotide platinum nanozyme [71]; (**B**) Nucleobase-templated copper nanomaterials [72]; (**C**) Ag_2_O NPs with laccase-like for malathion detection [74].

To overcome the intrinsic limitations of natural laccase in terms of pH, temperature, and storage, Wang et al. synthesized highly dispersed Pt nanoparticles using different oligonucleotides as stabilizers (including A10, T10, C10, and G10) and evaluated their catalytic activity in the oxidation of laccase substrates under ambient air [71]. The study demonstrated that Pt nanozymes possess broad temperatures and pH stability, and their laccase-like activity can be controlled by adjusting the sequence composition and the molar ratio of [precursor]/[template]. The C_10_-templated Pt nanozyme exhibited a three-fold higher affinity for 2,4-dichlorophenol than natural laccase (Figure 5A). This study lays the foundation for exploring the potential of artificial laccase-like nanozymes in biosensing.

Furthermore, Yang et al. developed a new strategy to modulate the laccase-like activity of copper nanomaterials using different DNA bases [72]. The study demonstrated that the cytosine-mediated copper nanozyme (C-Cu) exhibited the best laccase-like activity, and the enzyme activity could be further modulated by DNA sequences composed of multiple bases (Figure 5B). C-Cu displayed enhanced laccase-like catalytic activity, good thermal stability, and excellent oxidation capability, which enabled the efficient degradation and detection of phenolic pollutants in environmental media. Tang et al.’s study reported the first synthesis of a highly catalytic laccase-like nanozyme (Mn-GMPNS) with manganese ions as the active center and coordinated with guanosine monophosphate [73]. Compared with natural laccase, Mn-GMPNS exhibited excellent thermal stability, acid–base resistance, salt resistance, reusability, and substrate versatility. Huang et al. discovered that the laccase-like catalytic activity of the four base-pair cubic Ag_2_O NPs was significantly enhanced (Figure 5C) [74]. They hypothesized that the bases in the nucleotides might interact with the aromatic ring of the oxidized substrate 2,4-DP via secondary forces such as π-π stacking and hydrogen bonding. This interaction enables the aptamer to adsorb more substrate and move closer to the surface of the cubic Ag_2_O NPs, facilitating the generation of semiquinone free radicals.

### 3.6. GOx-like

**Figure 6:**
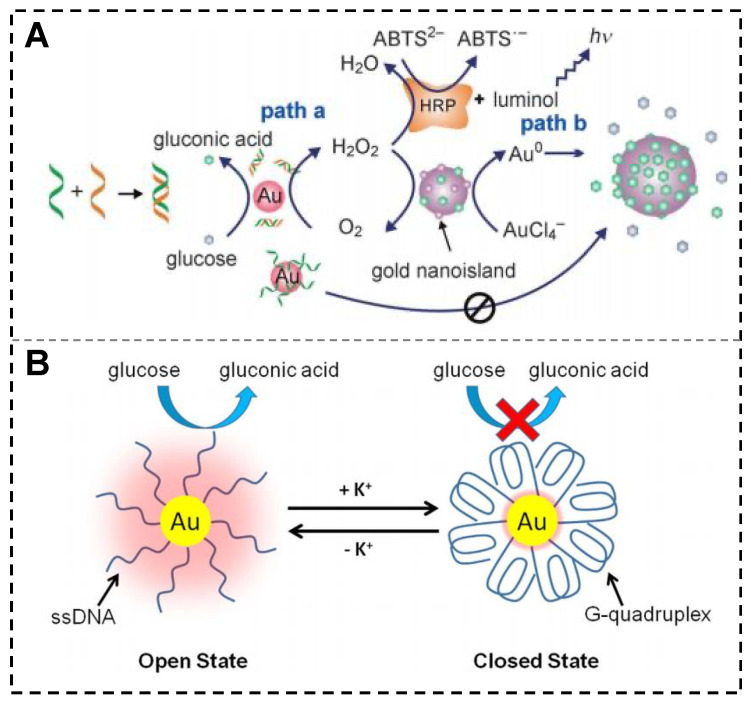
DNA-regulated GOx-like activity of AuNPs. (**A**) Changes in GOx-like activity of AuNPs upon interaction with ssDNA and dsDNA [76]. (**B**) Reversible regulation of AuNP catalytic activity by DNA nanomachines through cyclic K^+^ addition and removal [78].

GOx-like activity refers to the ability to catalyze the conversion of glucose to gluconic acid and H_2_O_2_ in the presence of O_2_. In 2004, Rossi and colleagues made the groundbreaking discovery that “naked” AuNPs synthesized without protectors or stabilizers exhibited GOx-like activity [75]. Zheng et al. investigated the changes in GOx-like catalytic activity of AuNPs upon interaction with ssDNA and dsDNA (Figure 6A) [76]. The results showed that ssDNA could be strongly adsorbed on the AuNPs surface, leading to the inactivation of the GOx-like catalytic activity. In contrast, the binding of dsDNA to AuNPs was weaker, only slightly interfering with the catalytic activity of AuNPs. The “naked” AuNPs without stabilizers have a short GOx-like activity lifetime, but the use of stabilizers and protectors (such as polyvinyl alcohol) inhibits the GOx-like activity. Zhang et al. found that AuNPs with natural protein (bovine serum albumin, BSA) as a stabilizer and protector exhibit GOx-like activity [77]. Furthermore, BSPP-AuNPs have improved stability in ionic solutions due to the presence of the BSPP protectant, but their GOx-like activity is reduced. When low-density ssDNA is immobilized on the surface of BSPP-AuNPs, it replaces a certain number of BSPP molecules, exposing the surface area around the DNA strands directly to the solution and activating their catalytic ability. K^+^ can promote the folding of G-rich DNA strands, and this structure has a diameter much larger than that of ssDNAs, resulting in a decrease in the contact area between glucose substrate molecules and the AuNPs surface, leading to deactivation of the catalyst (Figure 6B). Multiple cycles of regulation can also be achieved by alternately adding and removing K^+^, clearly demonstrating that the catalytic activity of AuNPs can be reversibly regulated by DNA nanomachines [78]. However, there are relatively few studies on nanozymes with GOx-like activity synthesized or regulated by nucleic acid templates, which requires further research.

Furthermore, nanozymes can be synthesized with two or even multiple enzyme-like activities by controlling the template type, composition, and metal doping. This not only enriches the types of nanozymes but also provides a basis for the cascade amplification of biosensor signals. For example, the bimetallic codoped CeO_2_ nanospheres (CoMn-CeO_2_ NSs) possess both POD-like and GOx-like activities, which were synthesized using a one-step hydrothermal method [79]. Studies have shown that the dual-enzyme activity enhancement of this nanozyme mainly originates from the doping of cobalt and manganese. The optimum pH for POD-like activity is 3.5, and the optimum pH for GOx-like activity is 7.4. Under the conditions of their respective optimum pH values, the other enzyme-like activity can be almost negligible. Therefore, by utilizing these two optimal pH values, CoMn-CeO_2_ NSs have the best activity while ensuring the relative independence of each signal transduction channel, which can improve the signal transduction efficiency. The NANs nanozymes mentioned above are detailed in Table 1.

## 4. Applications of NANs in Biosensors

### 4.1. Colorimetric Biosensors

NANs often utilize colorimetric methods for signal output, primarily due to their catalytic properties and the convenience and efficiency of colorimetric detection. Many of these nanozymes possess multiple enzymatic activities, enabling the oxidation of chromogenic substrates (such as TMB) in the presence of H_2_O_2_ to generate visible color changes. Colorimetric detection is straightforward, cost-effective, and highly sensitive, with the signal intensity measurable using a spectrophotometer. Furthermore, the design of NANs can be optimized to enhance catalytic performance and improve colorimetric reactions, making this approach suitable for bioanalysis, environmental monitoring, and medical diagnostics.

#### 4.1.1. Detection of Small Molecules

NANs demonstrate exceptional specificity and sensitivity in detecting small molecule toxins, neurotransmitters, redox substances, and other analytes. These nanozymes provide precise and reliable sensing capabilities, making them indispensable for applications in environmental monitoring, food safety, and medical diagnostics.

In 2022, a notable advancement was achieved with the synthesis of β-CD@DNA-CuNCs, which exhibit high POD-like activity. These nanozymes were synthesized using random DNA double strands as templates and β-cyclodextrin (β-CD) as a surface ligand. Based on β-CD@DNA-CuNCs as enzyme mimics, a simple and effective colorimetric method was developed for detecting glyphosate. Glyphosate disrupted the synergistic interaction of the redox couple (Cu^2+^/Cu^+^) on the β-CD@DNA-CuNCs surface, resulting in the inhibition of their POD-like activity. This inhibition influenced the catalysis of the TMB system, producing distinct colorimetric signals that enabled rapid and selective detection of pesticide residues. The method achieved a linear detection range of 0.02–2 μg/mL with an impressive limit of detection (LOD) of 0.85 ng/mL, highlighting its potential for high-sensitivity applications [80].

In mycotoxin detection, a colorimetric aptasensor based on a MOFzyme and HA-DNA hydrogel was developed. A Ce-Zr bimetallic MOF with OXD-like activity was synthesized via a thermosol process and partially oxidized. The MOFzyme surface was functionalized with trigger molecules and hairpin DNA structures for a hybridization chain reaction (HCR), with the hairpin structures linked to hyaluronic acid (HA) and incorporating ZEN-specific aptamer sequences. During the HCR, HA hydrogels gradually formed on the MOFzyme surface, inhibiting its OXD-like activity. In the presence of ZEN, the aptamer in the hydrogel bound to the target molecule, disrupting the hydrogel structure and causing its collapse. This restored the MOFzyme’s OXD activity, enabling TMB oxidation and producing a visible color change as the output signal. Under optimal conditions, the aptasensor exhibited a linear detection range of 0.001–200 ng/mL and a LOD of 0.8 pg/mL, offering a universal platform for the accurate quantification of food and environmental hazards [81].

In 2024, Zhu and colleagues developed a CuCo@PDA nanozyme-based aptamer-mediated lateral flow assay (Apt-LFA) platform for AFB1 detection. The CuCo@PDA nanozyme, with abundant functional groups, intrinsic dark coloration, and excellent POD-like activity, binds to the aptamer. Using a competitive sensing strategy, it generates a colorimetric signal on the test lines of the Apt-LFA. The biosensor achieved a LOD of 2.2 pg/mL. Recovery rates in real sample tests ranged from 95.11% to 113.77%, with coefficients of variation below 9.84%. The platform supports point-of-care testing (POCT) and quantitative detection via a smartphone-based device [82].

Due to their intrinsic oxidoreductase activity, NANs have a natural advantage in detecting small molecules with redox properties. In 2021, researchers demonstrated that DNA-copper hybrid nanoflowers, synthesized via the self-assembly of DNA and copper ions, exhibited significantly higher laccase-mimicking activity compared to materials synthesized without DNA. Further analysis revealed that hybrid nanoflowers composed of guanine-rich ssDNA and copper phosphate (GNFs) exhibited the highest catalytic activity, attributed to the favorable coordination between guanine and copper ions. Building on these findings, a paper-based microfluidic device was developed for the colorimetric detection of phenolic compounds. GNFs catalyzed the oxidation of phenolic compounds, producing products that reacted with 4-aminoantipyrine to form a colored adduct. The resulting colorimetric signal was conveniently quantified using a smartphone and ImageJ software (NIH). The paper’s microfluidic device exhibited excellent storage stability for up to two months and achieved spiked recovery rates ranging from 98.3% to 102.6%. These results highlight the potential of the GNF-based paper microfluidic device as an effective analytical tool for the convenient detection of phenolic compounds in POCT applications [83].

In 2023, Mei et al. investigated the regulatory effects of DNA oligonucleotides (A10, T10, C10, and G10) as nucleation templates on the POD-like activity of platinum nanozymes. They synthesized four types of platinum nanozymes with distinct POD activities (PtNP-A10, PtNP-T10, PtNP-C10, and PtNP-G10) and utilized them to construct a colorimetric sensor array capable of distinguishing six antioxidants: dopamine (DA), glutathione (GSH), AA, L-cysteine (L-Cys), uric acid (UA), and melatonin (MT). This sensor array could also quantitatively differentiate between various antioxidant concentrations and analyze mixed samples. Additionally, it demonstrated the ability to distinguish antioxidants in real-world samples, such as fetal bovine serum, underscoring its potential for practical applications [84].

**Figure 7:**
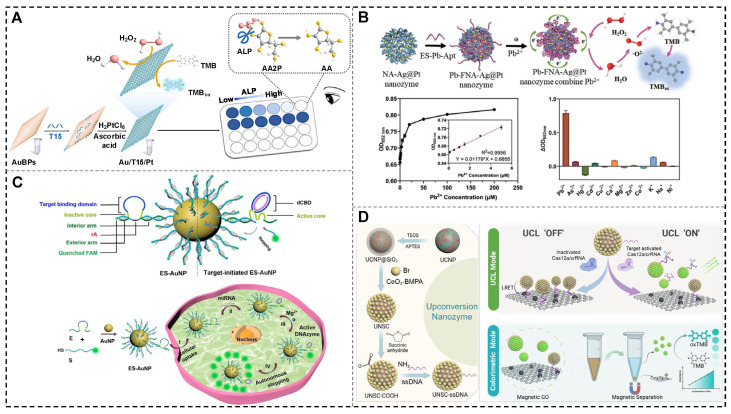
NANs for the construction of colorimetric and fluorescent sensors. (**A**) The bimetallic nanozyme for biosensing of AA and ALP [85]. (**B**) The Pb-FNA-Ag@Pt nanozyme for the construction of a Pb^2+^ aptasensor [8]. (**C**) The self-protected DNAzyme walker for miRNA imaging [98]. (**D**) The UCNP@SiO_2_@CeO_2_-ssDNA complex for the detection of target nucleic acids [99].

In the same year, Cai et al. developed a DNA-guided seed-growth strategy for synthesizing PtNPs on gold bipyramids (AuBPs), yielding a bimetallic nanozyme (Figure 7A). The synthesis was sequence-dependent, with the incorporation of a polyT sequence enabling the formation of nanostructures with significantly enhanced POD-like activity. As a proof of concept, the Au/T15/Pt nanozymes were used to design a simple and highly sensitive colorimetric assay for AA detection, achieving exceptional analytical accuracy. This work introduces a novel approach for the deliberate design of bimetallic nanozymes, expanding the potential applications of biosensing technologies [85].

NANs are widely employed for detecting H_2_O_2_ and glucose concentrations across various systems. In 2022, a novel approach utilizing DNA-based coordination-driven self-assembly was introduced for synthesizing an amorphous/crystalline hetero-phase nanozyme (Fe-DNA). This Fe-DNA nanozyme exhibited exceptional POD-like activity with minimal OXD-like activity, along with remarkable stability in its catalytic function, significantly improving the performance of nanozyme-based sensing platforms. The Fe-DNA nanozyme was successfully applied for the detection of H_2_O_2_ across a wide concentration range. Furthermore, it was used to develop a glucose sensor with outstanding sensitivity, selectivity, and reliability, highlighting its potential for advanced biosensing applications [9].

#### 4.1.2. Detection of Metal Ions

NANs are widely utilized for biosensing harmful metal cations, such as mercury (Hg^2+^) and lead (Pb^2+^). When integrated with paper-based or microfluidic devices, these systems are particularly suitable for POCT applications in food safety and environmental monitoring.

The detection of mercury ions is of critical importance, as mercury is a highly toxic pollutant that bioaccumulates in the food chain, posing severe risks to the human nervous, immune, and endocrine systems. Major sources of mercury include industrial emissions and environmental contamination [86,87]. Accurate and reliable detection methods are essential for monitoring pollution, ensuring food safety, and protecting ecological health, thereby mitigating the harmful impacts of mercury on both humans and the environment.

In 2020, a paper-based device was developed, consisting of a patterned paper chip, wicking pads, and a base. DNA-gold nanoparticles (DNA-AuNPs) were immobilized on the paper chip, where they interacted with Hg^2+^ ions to form DNA-AuNP/Hg^2+^ nanozymes capable of catalyzing the TMB-H_2_O_2_ reaction to produce a colorimetric signal. The wicking pads allowed for the application of larger sample volumes, enhancing the color response and improving detection sensitivity. The biosensor exhibited a linear detection range of 50–2000 nM for Hg^2+^, with a LOD of 10 nM. It demonstrated excellent performance in analyzing environmental water samples, achieving recovery rates of 85.7–105.6%. This cost-effective and user-friendly paper-based device holds significant promise for practical applications, particularly in environmental monitoring [88].

In 2022, researchers developed a DNA-encoded seed-growth method to synthesize MXene/DNA/Pt nanocomposites, which exhibited remarkable sequence-dependent POD-like activity. The activity of these nanocomposites was significantly inhibited in the presence of Hg^2+^ ions, which was attributed to the selective binding of Hg^2+^ and its in situ reduction to Hg^0^ at the hybrid interface. This nanozyme-based sensor demonstrated exceptional sensitivity, selectivity, and adaptability across a tunable dynamic range, making it highly effective for Hg^2+^ detection in both environmental and biological samples. The detection process could be performed using a UV-vis spectrophotometer, direct visual observation, or a microfluidic chip, underscoring its versatility and practicality for real-world applications [89].

For lead ion (Pb^2+^) detection, a one-step rapid self-assembly method driven by nucleic acid and metal ion coordination was recently employed to synthesize Ag@Pt nanozymes with exceptional POD-like activity and antibacterial properties (Figure 7B). By utilizing a C-rich sequence for nucleation and a G-rich sequence for activation, the FNA-Ag@Pt nanozyme achieved significantly enhanced catalytic performance. To address the substrate specificity limitations of conventional nanozymes, a Pb^2+^ aptamer was incorporated into the functional region of the nanozyme, resulting in the development of a Pb^2+^ aptasensor based on the Pb-FNA-Ag@Pt nanozyme. The sensor demonstrated a strong linear correlation between the Pb^2+^ concentration and OD_652 nm_ within the range of 0.1–5 µM, with a LOD of 21.7 nM, as determined using the 3σ rule. This sensitivity is well below the 72 nM maximum lead level set by the U.S. Environmental Protection Agency, highlighting its high potential for practical applications in environmental monitoring [8].

#### 4.1.3. Detection of Proteins

In recent years, the inherent recognition and catalytic functions of NANs have led to their growing application in the detection of protein targets, enabling precise measurement of both their concentration and activity [85,90,91].

Alkaline phosphatase (ALP), a vital hydrolase involved in phosphate metabolism, cellular regulation, and signal transduction, serves as a critical biomarker for diagnosing cardiovascular diseases, liver disorders, and bone-related ailments. Leveraging the high catalytic efficiency of Au/T15/Pt nanozymes, a colorimetric bioassay was developed to detect ALP activity. The assay is based on the inhibitory effect of AA on nanozymatic activity and the enzymatic conversion of ascorbic acid 2-phosphate (AA2P) to AA by ALP. This method exhibited a strong linear relationship between absorbance and ALP concentrations in the range of 0.625–5.5 mU/mL, with a LOD of 0.35 mU/mL. The bioassay also demonstrated excellent specificity, establishing it as a reliable and effective tool for analyzing ALP activity in biomedical applications [85].

#### 4.1.4. Detection of Whole Cells

NANs are also powerful tools for sensing and detecting microorganisms or whole cells [92]. In 2021, a DNA-encoded seed-growth method was developed to synthesize bimetallic dumbbell-like Au-Pt nanoparticles, representing the first exploration of DNA sequence-dependent growth in bimetallic nanomaterials. PolyT20 sequences facilitated the formation of dumbbell-shaped Au-Pt structures on gold nanorod seeds, while polyA20 and polyC20 produced similar structures but only at lower DNA concentrations due to their stronger binding affinity to metal surfaces. The synergistic interaction between gold and platinum in these nanoparticles significantly enhanced their catalytic activity as nanozymes. To detect *Escherichia coli* (*E. coli*) O157:H7, a thiolated aptamer targeting the bacteria was conjugated to the Au-Pt nano-dumbbells. A sandwich biosensor incorporating magnetic nanoparticles, bacterial cells, and the nanozyme was developed, achieving a broad linear detection range (10–10^7^ CFU/mL) and an exceptional LOD of 2 CFU/mL. This study advances the understanding of DNA-guided bimetallic nanoparticle synthesis and demonstrates its significant potential in biosensing and antimicrobial applications [93].

In 2022, Zhu et al. developed vancomycin-modified magnetic nanoparticles (Fe_3_O_4_@Au-Van MNPs) combined with octahedral Mn_3_O_4_ nanoparticles to construct a magnetic-assisted colorimetric biosensor. This biosensor utilized the specific binding capability of aptamers and the OXD-like activity of Mn_3_O_4_ nanoparticles for the highly sensitive and selective detection of *Staphylococcus aureus* (*S. aureus*) in complex samples. The SA31 aptamer acted as the recognition probe, and its interaction with the target bacteria modulated the OXD-like activity of Mn_3_O_4_ nanoparticles, producing a colorimetric signal. The biosensor demonstrated remarkable performance in detecting *S. aureus* in environmental and biological samples without requiring complex preprocessing. It exhibited excellent selectivity and sensitivity, achieving a LOD as low as 2 CFU/mL. Moreover, the Fe_3_O_4_@Au-Van MNPs and Mn_3_O_4_ nanoparticles retained high catalytic activity after multiple reuse cycles, providing a cost-effective and reliable approach for large-scale pathogen detection in industrial applications [94].

In 2024, Li et al. developed a colorimetric sensor for the detection of *E. coli* using a hydrothermal synthesis method to prepare an Fe_3_O_4_/MWCNTs@Mo-CDs nanozyme (FMMC), which exhibited excellent POD (POD)-like activity. The *E. coli* aptamer was conjugated to the FMMC surface, and the presence of the target bacteria modulated the nanozyme’s POD-like activity, generating a colorimetric signal. This straightforward and robust colorimetric aptasensor demonstrated a wide linear detection range of 10–10^6^ CFU/mL, a low limit of quantification (LOQ) of 10 CFU/mL, and a LOD of 0.978 CFU/mL. The method exhibited excellent selectivity and achieved high recovery rates in real samples, including milk, grape juice, and orange juice, highlighting its potential for practical applications in food safety monitoring [95].

Studies have also employed NANs for cancer cell detection, showcasing their potential in biomedical applications. In 2021, Wei et al. developed novel nanoflower-shaped photothermal nanostructures via a one-pot metallization-like synthesis using polyadenine-containing diblock DNA as a scaffold. This bifunctional DNA consisted of a polyadenine block and a recognition block (AS1411 aptamer). The resulting nanoflower-shaped nanozymes exhibited high enzymatic activity and enabled the specific recognition and colorimetric sensing of cancer cells. With the integration of 808 nm laser irradiation, the sensor achieved exceptional sensitivity and selectivity, detecting cancer cells at a limit as low as 10 cells/mL. This strategy highlights the potential of nanozyme-based colorimetric sensors for POCT applications, advancing the development of rapid and reliable diagnostic tools [96].

#### 4.1.5. Detection of Nucleic Acids

Using NANs for nucleic acid target detection represents an innovative and promising strategy. In 2020, Guo et al. introduced a refined approach to modulate the catalytic activity of DNA-templated silver nanoclusters (DNA-AgNCs) through the unique adsorption behavior of DNA on DNA-AgNCs and the reversible transitions between double-stranded and triple-stranded DNA structures. The study demonstrated that four DNA homopolymers exert distinct regulatory effects on the catalytic activity of DNA-AgNCs. Notably, the formation of T-A•T triplex DNA restored the catalytic activity of DNA-AgNCs from its deactivated state caused by ssDNA or dsDNA. This restoration was attributed to the interaction of adenine’s N7 groups with DNA-AgNCs, which blocks active sites. Based on this mechanism, the catalytic activity of DNA-AgNCs could be reversibly regulated via DNA input-triggered transitions between duplex and triplex DNA structures. Leveraging this reversible regulation, the researchers developed two cost-effective and straightforward biosensing methods for detecting DNA targets, providing a novel and versatile approach to nucleic acid biosensing [97].

NANs, characterized by their high programmability and versatility, offer significant potential for advancing colorimetric sensors. Their ability to catalyze chromogenic reactions, coupled with specific recognition and signal output capabilities, has demonstrated exceptional performance in bioanalysis, environmental monitoring, and medical diagnostics. Future developments will focus on enhancing the stability of the reactions and the strength of the signals to meet the detection requirements in various environmental conditions.

### 4.2. Fluorescent Biosensors

NANs are extensively utilized in fluorescence sensors, exploiting the fluorescent properties of inorganic nanomaterials or nucleic acid molecules for signal output. Through the specific recognition of target molecules and the induction of fluorescence changes, these methods deliver high sensitivity and specificity, making them particularly effective for on-site POCT. In addition, NANs offer unique advantages in in vivo nucleic acid imaging and disease diagnosis. They facilitate real-time tracking of biomolecular dynamics, providing powerful tools for precision medicine and early disease screening. These attributes underscore their significant potential for diverse applications in medical diagnostics and beyond.

#### 4.2.1. Detection of Small Molecules

As for the biosensing of small molecules, Wang et al. (2024) introduced a nanozyme-based ratiometric fluorescent platform for detecting cysteine (Cys) and bleomycin (BLM). This system utilizes a cost-effective “mix-and-act” G-quadruplex/Cu(II) (G4/Cu) metal nanozyme with excellent POD-like activity. The detection mechanism is based on the catalytic oxidation of two fluorescent substrates by the G4/Cu nanozyme in the presence of H_2_O_2_, where the substrates exhibit opposite fluorescence responses. The specific interaction between Cu^2+^ and the target molecules enables highly sensitive detection, with a strong linear relationship observed between the emission peak ratio of the two fluorescent dyes and the target concentration. The sensor achieved LODs of 6.7 nM for Cys and 10 nM for BLM, highlighting its potential for precise and efficient small molecule detection [100].

#### 4.2.2. Detection of Metal Ions

NANs have demonstrated significant potential in metal cation sensing by combining the specific recognition and cleavage properties of metal ion-dependent DNAzymes with the efficient catalytic activity of nanozymes. This approach enables highly sensitive and selective detection by triggering signal output through the interaction of metal ions with DNAzymes [101,102]. NANs have demonstrated significant potential in metal cation sensing by combining the specific recognition and cleavage properties of metal ion-dependent DNAzymes with the efficient catalytic activity of nanozymes. This approach enables highly sensitive and selective detection by triggering signal output through the interaction of metal ions with DNAzymes [103,104,105].

Yang et al. developed a Zn^2+^-specific near-infrared (NIR) DNAzyme nanoprobe for the real-time tracking of metal ions with spatiotemporal precision in zebrafish embryos and larvae. This approach employed photocaged DNAzymes conjugated to lanthanide-doped UCNPs. The UCNPs converted deeply penetrating NIR 980 nm light into 365 nm emissions, allowing the UV photons to efficiently uncage the substrate strand and enable enzymatic cleavage by a Zn^2+^-specific DNAzyme. Upon cleavage, the released product contained a visible fluorophore that was initially quenched, generating a detectable fluorescent signal. The DNAzyme-UCNP probe effectively sensed Zn^2+^ within the NIR biological imaging window and enabled visible detection in both living cells and zebrafish embryos, offering a powerful tool for precise metal ion tracking in complex biological environments [104].

A magnetic DNAzyme nanomachine-based fluorescent biosensor was constructed recently for rapid and ultra-sensitive detection of Pb^2+^. The design employed BSA as a linker to connect magnetic beads (MBs) with DNAzyme, significantly enhancing detection sensitivity. In the presence of Pb^2+^, the DNAzyme’s enzymatic activity was activated, resulting in the cleavage and release of substrate strands. Pb^2+^ further facilitated a cyclic recognition and release process, amplifying the detection signal. The cleavage products and stem-loop chains were subsequently amplified through a double-primer real-time fluorescence quantitative reaction, enabling fluorescence signal monitoring. Under optimized conditions, the biosensor exhibited a linear detection range of 2 nM to 150 nM and achieved a LOD of 3.7 nM for Pb^2+^. This creative biosensor provides a highly sensitive and efficient tool for Pb^2+^ detection [105].

#### 4.2.3. Detection of Proteins

Tumor-specific molecular imaging allows for the real-time and precise identification and localization of specific biomarkers within the tumor microenvironment. This approach is essential for early tumor detection, staging assessment, and therapeutic monitoring, significantly improving the accuracy and effectiveness of cancer diagnosis and treatment [106,107].

In 2023, Wang et al. developed an endogenous APE1-activated autonomous-motion DNAzyme signal amplification strategy for tumor-specific molecular imaging. The system utilizes three hybridized DNA strands immobilized on AuNPs via Au-S bonds, forming an enzyme-activated DNAzyme probe (E-DNAzyme). Fluorescence is initially quenched through fluorescence resonance energy transfer (FRET) between FAM and the AuNPs. In the presence of APE1, enzymatic cleavage induces conformational changes in the E-DNAzyme, restoring fluorescence and providing a direct reflection of APE1 levels. The E-DNAzyme operates in a recycling mode, leveraging SDA to further enhance the signal. This design significantly improves sensitivity and spatial specificity for tumor-specific molecular imaging, offering a robust tool for cancer diagnostics and therapeutic monitoring [108].

#### 4.2.4. Detection of Whole Cells

Microbial monitoring is essential for protecting public health, ensuring food safety, maintaining environmental quality, and optimizing industrial production. In 2020, Zhou et al. developed a fluorescent sensor with triple signal amplification, utilizing MBs, DNAzyme, and photoluminescence for detecting *E. coli*. This method employs an E. coli-specific DNAzyme that selectively recognizes the target protein in crude intracellular mixtures, triggering conformational changes to initiate RCA. RCA subsequently produces CuNCs, generating a luminescent signal. The biosensor achieved a strong linear detection range of 10–1000 CFU/mL with a LOD of 1.57 CFU/mL. It also demonstrated rapid detection within 1.5 h, high efficiency, and excellent reproducibility, making it suitable for detecting *E. coli* O157:H7 in drinking water and apple juice samples [109].

NANs offer substantial potential in the development of fluorescent sensors. By combining the high specificity of nucleic acid recognition with the catalytic efficiency of nanozymes, these systems enable sensitive and selective signal output via fluorescence dyes, luminescent materials, or FRET mechanisms. They are widely applied in disease diagnosis, environmental monitoring, and food safety. Future research will focus on enhancing the fluorescence signal output of NANs, ensuring high sensitivity even at low concentrations. Additionally, researchers may explore the design of multicolor fluorescence labels, enabling multiplexed detection for broader applications, particularly in early disease screening and clinical diagnosis.

#### 4.2.5. Detection of Nucleic Acids

NANs have demonstrated significant potential in the detection of miRNA and other nucleic acid molecules. By integrating the high specificity of nucleic acid recognition with the efficient catalytic properties of nanozymes, these systems enable signal output through target-induced conformational changes or catalytic reactions. NANs exhibit exceptional sensitivity and selectivity, even in low-concentration or complex environments, making them highly effective for nucleic acid detection. Their versatility holds considerable value in applications such as biomarker detection, early disease diagnosis, and gene expression analysis, providing powerful tools to advance precision medicine and molecular diagnostics.

miRNA plays a critical role in regulating gene expression and influencing essential cellular processes, including proliferation, differentiation, and apoptosis. Abnormal miRNA expression is closely associated with tumorigenesis and disease progression, establishing miRNA as a crucial biomarker for cancer diagnosis, prognosis, and therapeutic monitoring [110,111]. In recent years, DNAzyme-based nanozymes have been extensively used for miRNA detection and imaging [98,112,113]. As shown in Figure 7C, researchers developed a self-protected DNAzyme (E) walker for imaging intracellular miRNA, using miRNA-21 as a model target. By integrating a circular bulging DNA shield into the catalytic core, the DNAzyme walker achieved full interaction with the substrate (S)-modified AuNP, enabling efficient imaging. This self-protected DNAzyme walker demonstrated high efficiency and durability in miRNA imaging within living cells and mice, providing a valuable platform for detecting various miRNAs in live-cell and in vivo environments. This approach holds significant promise for advancing miRNA research and applications in biological systems [98].

In 2023, Huang et al. developed a DNAzyme-based dual-feedback autocatalytic exponential amplification biocircuit supported by a honeycomb MnO_2_ nanosponge (EDA2@hMNS) to enable live-cell imaging of low-abundance intracellular miRNAs. The EDA2 biocircuit integrates a blocked DNAzyme (b-DNAzyme), a fuel strand, and a substrate strand, enabling efficient signal amplification. This system recycles target miRNAs and generates miRNA analogues through DNAzymatic reactions, establishing a dual-feedback mechanism that drives multiple parallel cascade amplifications. As a result, the EDA2@hMNS achieved a LOD of 17 pM, improving sensitivity by 288-fold compared to the b-DNAzyme alone [113].

DNA methylation is a crucial epigenetic regulatory mechanism that governs gene expression, genome stability, and cellular differentiation. Aberrant DNA methylation is strongly associated with various diseases. Detecting DNA methylation provides insights into functional changes in specific genes or genomic regions, contributing to early diagnosis, prognosis, and personalized treatment [114,115]. In 2023, Yang et al. developed oxygen-functionalized polypyrrole quantum dots (o-ppy QDs) with exceptional POD activity under mild conditions. These QDs exhibited superior catalytic efficiency, outperforming oxidized graphene QDs and demonstrating activity 120 times higher than horseradish peroxidase (HRP). Guanine (G) and adenine (A) bases displayed strong binding affinities to o-ppy QDs, with G significantly enhancing catalytic activity and A reducing it. This finding enables precise, sequence-dependent regulation of catalytic activity by designing specific DNA sequences. Based on these properties, a dual-mode colorimetric and fluorescent biosensor was developed for DNA methylation detection, achieving a LOD of 2 × 10^−9^ M. This novel approach represents a powerful tool for advancing epigenetic research and diagnostic applications [116].

As shown in Figure 7D, Yan et al. recently developed a dual-mode CRISPR-Cas12a biosensing platform that integrates upconversion luminescence resonance energy transfer (LRET) with nanozyme-based colorimetric detection for nucleic acid analysis. This system employs a functionalized nanocomposite (UNSC-ssDNA@MGO), where upconversion nanozymes (UCNP@SiO_2_@CeO_2_-ssDNA, abbreviated as UNSC-ssDNA) are adsorbed onto Fe_3_O_4_-composited MGO nanosheets through π-π interactions between ssDNA and MGO. In this platform, upconversion nanoparticles (UCNPs) act as upconversion luminescence donors, CeO_2_ serves as the nanozyme, and MGO functions as both a luminescence quencher and a magnetic separator. Upon the introduction of target nucleic acids, Cas12a cleaves the ssDNA on the UNSCs, causing them to detach from the MGO surface. This detachment restores luminescence and releases the nanozyme, resulting in a colorimetric change in the solution. This dual-mode biosensor enables rapid and highly sensitive nucleic acid detection with a fluorescence LOD of 320 fM [99].

### 4.3. Electrochemical Biosensors

Electrochemical sensors, which leverage redox reactions or signals such as current and potential, enable highly sensitive detection of target analytes. These sensors offer key advantages, including rapid response, high sensitivity, and low detection limits, making them valuable tools in biomarker detection, environmental monitoring, and food safety.

#### 4.3.1. Detection of Small Molecules

NANs are extensively utilized in electrochemical sensors for small molecule detection, leveraging their catalytic properties for the direct conversion of electrochemical signals. This approach often eliminates the need for complex labeling processes, enabling real-time monitoring of current, potential, or impedance changes to rapidly quantify target small molecule concentrations. Compared to traditional detection methods, electrochemical sensors offer notable advantages, including simplicity, high sensitivity, and portability. Their ability to directly translate molecular interactions into measurable signals makes them highly effective for practical applications [117,118]. These features make them an efficient and practical tool for the detection of small molecules.

Aflatoxin B1 (AFB1), the most toxic and harmful aflatoxin, poses a severe threat to food safety and public health. In 2023, a dual-mode microfluidic paper-based analytical device (μPAD) was developed for AFB1 detection, integrating both electrochemical and colorimetric readouts. The device utilized AuNPs supported on Ni-Co layered double hydroxide nanocages (Au/Ni-Co LDH NCs) as signal amplifiers. Tetrahedral DNA nanostructures (TDNs) served as scaffolds to enhance the binding efficiency of “bottom-up” aptamers for capturing AFB1. The Au/Ni-Co LDH NCs, exhibiting excellent POD-like activity, were attached to TDNs via hybridized aptamers and complementary DNA (cDNA), producing a strong electrochemical signal through H_2_O_2_ reduction. This sensor demonstrated a wide detection range (0.2 pg/mL to 100 ng/mL) and an ultra-low LOD of 0.071 pg/mL, showcasing its potential for highly sensitive and reliable AFB1 detection [119].

Aminoglycoside antibiotics are a class of broad-spectrum antibacterial agents. In 2024, a composite material combining Au-Pd bimetallic nanoparticles with ferrocene was developed, utilizing the catalytic and electrochemical properties of ferrocene’s cyclopentadiene and carboxyl groups to bind amino-modified DNA sequences, forming signal probes. In the presence of target molecules, exonuclease-assisted cyclic amplification increased the density of signal probes on the electrode surface, enhancing electron transfer and altering the electrical signal. A linear correlation was observed between the logarithm of the AA concentration and the current in the range of 0.1–1000 nM, with a LOD of approximately 0.0355 nM. This study provides valuable insights into the design of multi-mode aptamer-based sensors for antibiotic detection [120].

**Figure 8:**
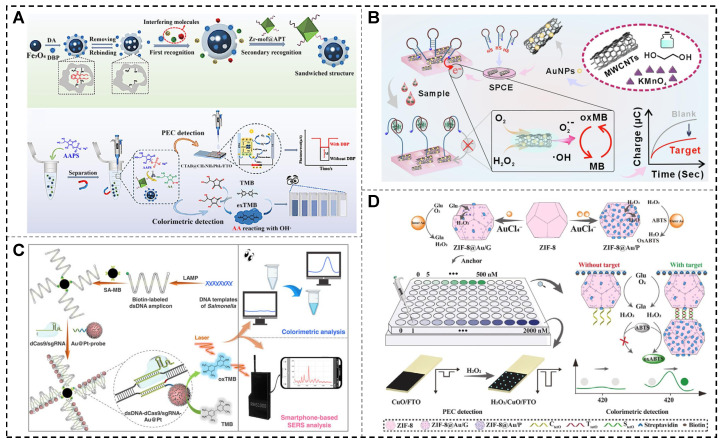
NANs for the construction of electrochemical, Raman, and chemiluminescent sensors. (**A**) The Zr-MOF@Apt-DBP-Fe_3_O_4_@MIPs sandwich structure used for DBP detection [121]. (**B**) The MWCNTs/MnO_2_/Au nanozyme used for the detection of tau protein [130]. (**C**) The Au@Pt nanozyme used for the construction of a microbial biosensor [140]. (**D**) The dumbbell-shaped cascade nanozyme used for the dual-mode visual and PEC detection of antibiotic resistance genes (ARGs) [146].

In 2024, Shen et al. developed a dual-mode photoelectrochemical (PEC)-colorimetric sensing platform based on a molecularly imprinted polymer (MIP)-aptamer sandwich structure and nanoenzymes (Figure 8A). The platform incorporates Fe_3_O_4_@MIPs, which act as POD mimics, and Zr-MOF@Apt, a zirconium-based metal–organic framework labeled with aptamers that mimic ALP, as key recognition elements. Dibutyl phthalate (DBP) facilitates the formation of a Zr-MOF@Apt-DBP-Fe_3_O_4_@MIPs sandwich complex, allowing magnetic separation and enzymatic reactions to generate both electrochemical and colorimetric signals. In the electrochemical mode, the sensor demonstrated a broad detection range of 1.0 pM to 10 μM and achieved a LOD of 0.263 nM. By combining dual-signal output with a sandwich recognition strategy, this platform offers enhanced sensitivity and precision, making it a promising tool for detecting small molecules in environmental and food safety applications [121].

#### 4.3.2. Detection of Metal Ions

Mercury ions (Hg^2+^) are highly toxic heavy metal pollutants, and their detection is vital for environmental protection, food safety, and human health [122,123]. In 2022, Wang et al. developed an ultrasensitive electrochemical aptasensor for Hg^2+^ detection, utilizing gold-modified thiol graphene (Au@HS-rGO) as the sensing platform and gold–palladium-modified zirconium metal–organic frameworks (AuPd@UiO-67) as signal amplifiers. Nucleic acid chains served as recognition elements for specific detection. The aptasensor exhibited a wide detection range of 1.0 nM to 1.0 mM and a LOD of 0.16 nM under optimal conditions. It demonstrated excellent selectivity, reproducibility, and stability, along with outstanding performance in real water sample analysis. This study highlights the potential of this aptasensor for precise and reliable mercury ion monitoring in environmental applications [124].

#### 4.3.3. Detection of Proteins

NANs are employed in electrochemical sensors for protein detection, often utilizing aptamers as recognition elements. These biosensors enable highly sensitive signal output, offering an efficient and reliable method for protein analysis. Cardiac troponin I (cTnI) is a highly specific biomarker for myocardial injury. Its detection is crucial for diagnosing, monitoring therapy, and predicting outcomes in acute myocardial infarction (AMI) and other cardiovascular diseases [125,126]. In 2019, Sun et al. developed an electrochemical dual-aptamer biosensor for the detection of cTnI, a key biomarker for myocardial injury. This biosensor utilized DNA nanotetrahedron (NTH)-based Tro4 aptamer probes and multifunctional nanoprobes to achieve enhanced signal amplification and detection sensitivity. The thiolated NTH-Tro4 capture probes were immobilized onto a screen-printed gold electrode (SPGE) via thiol–gold bonding, creating a biomimetic interface that significantly improved cTnI recognition. Multifunctional nanoprobes, incorporating the Tro6 aptamer, HRP, and HRP-mimicking gadolinium-doped hollow nanospheres (GHD) immobilized on Fe_3_O_4_/Au@Pt hybrid nanozymes, facilitated the formation of an NTH-Tro4/cTnI/nanoprobe sandwich structure upon target binding. This design enhanced the biosensor’s sensitivity and specificity for cTnI detection [127].

Tau protein is a critical pathological biomarker for Alzheimer’s disease (AD) and other neurodegenerative disorders, playing a pivotal role in early diagnosis, monitoring disease progression, and evaluating therapeutic efficacy [128,129]. As shown in Figure 8B, Chen et al. (2024) developed a high-sensitivity, one-step chronocoulometric method for detecting tau protein in clinical samples. This approach utilized a multi-enzyme mimic nanozyme (MWCNTs/MnO_2_/Au) to enhance the catalytic performance of methylene blue as a signal generator. The detection mechanism involved a hairpin aptamer probe that underwent conformational changes upon binding to tau protein, producing measurable electrical signals. The MWCNTs/MnO_2_/Au nanozyme exhibited excellent catalytic activity, achieving a LOD of 0.3 pg/mL for tau protein. The sensor demonstrated outstanding accuracy in plasma samples spiked with tau protein, with recovery rates ranging from 88.90% to 105.51%. This advanced platform provides a precise and reliable solution for tau protein detection, highlighting its potential for clinical diagnostics and AD research [130].

#### 4.3.4. Detection of Whole Cells

In recent years, functional NAN-based electrochemical sensors have been increasingly applied for the detection of cancer cells and pathogenic microorganisms. In 2022, a highly sensitive electrochemical cytosensor was developed using a triple signal amplification strategy. Fe_3_O_4_@Au nanozymes and DNAzyme hybrids were utilized as nanoprobes, with toluidine blue (Tb) serving as an electron transfer mediator. The Fe_3_O_4_@Au nanocomposites acted as efficient nanozymes for H_2_O_2_ reduction and provided scaffolds for loading electroactive substances and DNA probes. The DNA probes were designed with dual functionalities: a hemin/G-quadruplex sequence functioning as a DNAzyme and an aptamer sequence for the specific recognition of cancer cells. Tb, integrated into the Fe_3_O_4_@Au hybrids, further amplified the electrochemical response by enhancing electron transport. This triple amplification strategy significantly improved the sensor’s sensitivity. The cytosensor exhibited exceptional detection performance for HepG2 cells, achieving a LOD of 20 cells/mL, underscoring its potential for early cancer diagnosis and clinical applications [131].

In 2024, Wang et al. developed a novel porous hydrogel material (Au@PEI-ABEI@Pt) for an ultrasensitive electrochemiluminescence (ECL) assay to detect *Burkholderia pseudomallei*. The hydrogel, composed of polyethyleneimine-luminol (PEI-ABEI), AuNPs, and PtNPs, features a porous structure with a large surface area, enhancing its functional properties. By integrating this hydrogel with an ECL system and a CRISPR/Cas12a signal amplification strategy, the platform achieved exceptional sensitivity, with a LOD of 5 CFU/mL in complex samples. It demonstrated high specificity and stability, effectively distinguishing *Burkholderia pseudomallei* from other Gram-negative bacteria. This study presents a novel application of CRISPR/Cas systems combined with solid-phase carriers, providing a powerful tool for microbial detection and advancing diagnostic technologies [132].

#### 4.3.5. Detection of Nucleic Acids

NAN-based electrochemical sensors are highly effective for detecting target nucleic acid molecules. In 2020, Li et al. developed an electrochemical biosensor that combined a cascade primer exchange reaction (PER) with a MOF@Pt@MOF nanozyme for the highly sensitive detection of exosomal miRNA. The PER system, composed of a gated hairpin, a primer, and DNA polymerase, autonomously generates long ssDNA under isothermal conditions. This process releases protector B, which unblocks capture probes, allowing the nanozyme to bind to the sensing surface and produce an amplified electrochemical signal via its catalytic activity. The biosensor demonstrated exceptional sensitivity, with a LOD of 0.29 fM, and high specificity, effectively distinguishing homologous miRNAs with single-base mismatches. It successfully identified tumor cells and breast cancer patients by detecting exosomal miRNA-21, with results closely matching qRT-PCR analysis [133].

NANs offer distinct advantages in the design of electrochemical sensors. By combining molecular recognition with catalytic capabilities, they efficiently translate recognition events into electrochemical signals, enabling the detection of a wide variety of targets, including small molecules, metal ions, and proteins. In terms of electrochemical output, future research will focus on improving the stability of NANs in complex environments and their durability during long-term operation. At the same time, by integrating miniaturization technologies with portable devices, the application of electrochemical sensors will become more widespread, particularly showing tremendous market potential in remote monitoring and point-of-care diagnostics.

### 4.4. SERS Biosensors

The integration of surface-enhanced Raman scattering (SERS) technology with nanozymes significantly enhances detection sensitivity, specificity, and reliability. SERS provides ultra-high signal amplification, enabling single-molecule-level detection, while nanozymes, with their high catalytic activity, amplify target signals, achieving a dual signal-enhancement effect. This combination also reduces non-specific interference, improving detection accuracy and supporting multiple detection modes, including colorimetric, fluorescence, and electrochemical methods, thereby enhancing data validation. This integrated technology holds great potential in bioanalysis, environmental monitoring, disease diagnosis, and food safety, overcoming the limitations of single detection methods and enabling more efficient and precise analysis of complex samples [134].

NAN-based SERS sensors are highly sensitive and specific detection platforms that combine the molecular recognition capabilities of nucleic acids, the catalytic efficiency of nanozymes, and the signal enhancement properties of gold or silver substrates. By integrating nucleic acids with nanozymes or metallic inorganic materials, these sensors can specifically identify target molecules, including small molecules, proteins, or nucleic acids. Nanozyme-catalyzed reactions amplify the signal, significantly enhancing the SERS response of the target. The gold or silver substrate further boosts sensitivity through strong localized surface plasmon resonance effects, enabling the efficient detection of trace amounts of target analytes [135].

Histamine (HA), a bioactive amine widely present in the human body and various foods, plays essential roles in regulating allergic reactions, gastric acid secretion, and neurotransmission. However, excessive intake or abnormal metabolism of HA can pose significant health risks, emphasizing the need for its accurate detection in food safety, medical diagnostics, and industrial production [136,137]. In 2023, Wang et al. developed a highly sensitive SERS biosensor for HA detection using aptamers conjugated to AuNPs. This bioconjugation enhanced the nanozyme’s POD-like activity. In the presence of HA, the target molecule binds to its aptamer, causing the aptamer to detach from the AuNP surface. This detachment reduces the nanozyme’s catalytic activity, which is quantified through the TMB-H_2_O_2_ system. The sensor demonstrated exceptional performance, achieving a broad linear detection range of 10^−11^ to 10^−3^ M and an impressive LOD of 1.22 × 10^−12^ M [138].

To achieve a more advanced design, Ma et al. (2024) developed a SERS aptasensor for HA detection. The sensor utilized MIL-100(Fe) loaded with AuNPs to form a composite nanozyme (MIL-100(Fe)@AuNPs) with high catalytic efficiency for the TMB/H_2_O_2_ reaction. Silver nanoparticles (AgNPs) were synthesized as signal amplifiers to enhance the SERS signal of oxTMB. These components were functionalized with nucleic acids and assembled into a multifunctional substrate combining catalytic and SERS-enhancing properties. During detection, the specific binding of the aptamer to HA inhibited the assembly of AgNPs on MIL-100(Fe)@AuNPs, resulting in a decrease in oxTMB SERS signals. The sensor exhibited a broad linear detection range of 10^−11^ M to 5 × 10^−3^ M and an ultralow LOD of 3.9 × 10^−12^ M. Practical applicability was validated by recovery rates of 94.42% to 105.75% in fermented soybean products. This advanced SERS aptasensor offers a precise and effective tool for HA monitoring, supporting food safety during processing and storage [139].

A novel platform for microbial detection was developed, leveraging dCas9 to specifically recognize repetitive sequences in LAMP-generated amplicons (Figure 8C). This interaction formed nucleic acid frameworks that organized bifunctional gold–platinum (Au@Pt) nanozymes into chains on streptavidin-magnetic beads (SA-MB). The Au@Pt nanozymes catalyzed the conversion of colorless TMB into blue oxTMB via their platinum shell, while the gold core enhanced the Raman signal of oxTMB. This dual-mode system enabled both colorimetric and SERS detection of *Salmonella*, achieving remarkable sensitivity with a LOD of 1 CFU/mL within 50 min. The platform’s effectiveness was validated in various real samples, including lake water, milk, orange juice, beer, cabbage, and eggs [140].

NAN-based SERS sensors have proven highly effective for miRNA detection. Li et al. developed a system where the presence of miRNA induces nucleic acid hybridization, enabling the selective self-assembly of gold nanospheres onto hollow Au/Ag alloy nanocuboids. These nanostructures were engineered with optimal interparticle distances (~2.3 nm) to maximize SERS signal enhancement. The self-assembled nanostructures, coupled with nanozyme-catalyzed SERS signaling cascades, facilitated highly sensitive miRNA detection, achieving a LOD in the fM range for miR-107 within clinically relevant levels without requiring molecular amplification. Validation with clinical samples highlighted the potential of miR-107 as a non-invasive biomarker for prostate cancer diagnosis. This strategy demonstrates the promise of nanozyme-based SERS sensors for sensitive, precise, and amplification-free miRNA detection in clinical applications [135].

NANs in SERS sensors combine the high specificity of aptamers with the signal enhancement capabilities of gold or silver substrates to achieve the ultrasensitive detection of targets, including small molecules, nucleic acids, and microorganisms. These sensors translate specific biomolecular interactions and nanozyme-catalyzed amplification into strong Raman signals, offering a rapid response, high sensitivity, and excellent anti-interference capabilities. Future research will aim to optimize the synergy between nucleic acids and nanomaterials to further improve signal amplification efficiency and detection stability. Additionally, expanding the application potential of these sensors for analyzing complex samples will be a key focus, enhancing their utility in diverse fields.

### 4.5. Other Biosensors

NANs are often integrated with chemiluminescent signal output methods for detecting nucleic acid and non-nucleic acid targets. This approach utilizes the catalytic properties of nanozymes to amplify chemiluminescent signals triggered by specific target interactions, enabling sensitive and efficient detection [141,142,143]. In 2024, Qiu et al. developed a catalytically competent supramolecular system, termed the assembly activated hemin enzyme (AA-heminzyme), by combining hemin, histidine analogs, and G-quadruplex DNA. This system exhibited remarkable catalytic performance, chemiluminescent capability, chemical stability, reusability, and cost-effectiveness. Additionally, its design allows easy modification to introduce new functionalities. The AA-heminzyme demonstrated high efficiency and sensitivity in detecting GSH via a chemiluminescent mode, achieving results comparable to commercial kits while significantly reducing costs. This innovative system highlights the potential of AA-heminzyme for practical applications in cost-effective and sensitive target detection [142].

NANs integrated with PEC technology have enabled the development of innovative and highly efficient sensing platforms. These platforms hold significant potential in biomedicine, environmental monitoring, and food safety. PEC sensors utilize light energy to drive electrochemical reactions, allowing highly sensitive target detection by converting light and electrical signals [144,145]. In 2024, a dual-mode biosensor was designed using CdIn_2_S_4_ hollow microspheres as signal probes for ECL and PEC, combined with Au@CuO/Cu_2_O nanozymes for detecting Sa-16S rDNA. CdIn_2_S_4_ microspheres provided strong PEC and ECL signals, while an Exo-III amplification strategy enabled the introduction of Au@CuO/Cu_2_O nanoparticles to the electrode. These nanozymes, with efficient POD-like activity, generated signals proportional to the target concentration. This multifunctional biosensor exemplifies the effective integration of CdIn_2_S_4_ and nanozymes, offering a flexible and highly sensitive platform for DNA detection. It provides valuable insights into advancing nanozyme-based PEC biosensing technologies for molecular diagnostics and related applications [143].

Recently, Yao et al. designed a dumbbell-shaped cascade nanozyme for the dual-mode visual and PEC detection of ARGs, as shown in Figure 8D. This creative system features AuNPs anchored on ZIF-8 dodecahedrons, which exhibit GOx-like activity (ZIF-8@Au/G) and POD-like activity (ZIF-8@Au/P). In the presence of ARGs, DNA hybridization triggers the formation of an asymmetric dumbbell-like structure. One end incorporates ZIF-8@Au/G with captured DNA, while the other end includes ZIF-8@Au/P with signal DNA, enabling a cascade amplification process. This configuration facilitates highly efficient colorimetric and PEC detection, achieving a LOD of 0.112 nM. The bioassay was successfully validated by detecting ARGs in real sludge samples, highlighting its applicability for environmental monitoring and its potential for the sensitive detection of ARGs in complex systems [146].

NANs integrated with photothermal sensor technology provide a novel and efficient platform for precise analyte detection. These sensors combine the target recognition capabilities of functional nucleic acids with the photothermal conversion properties of nanozymes, enabling sensitive detection through heat-based signal transduction [147,148,149]. In 2024, Yu et al. developed a dual-mode biosensing platform combining fluorescence and photothermal detection. This system integrates catalytic hairpin assembly (CHA), toehold-mediated strand displacement reaction (SDR), and a DNA walking machine, along with dual identification and signal reporting modules governed by an AND logic gate and MBs. In the presence of bispecific miRNAs, the AND logic gate activates, initiating the DNA walking machine to collect hairpin DNA strands labeled with FAM fluorophores and CeO_2_@Au nanoparticles. These nanoparticles act as nanozymes, catalyzing the oxidation of TMB into oxTMB and producing a NIR photothermal effect after MB separation. This versatile biosensing platform successfully distinguished plasma samples from breast cancer patients, lung cancer patients, and healthy donors, demonstrating its potential for clinical diagnostics and disease differentiation [150].

NANs have been successfully integrated with various signal output technologies, including chemiluminescence, PEC, and photothermal sensing, to develop innovative and highly sensitive biosensing platforms. By combining the catalytic properties of nanozymes with the target recognition capabilities of nucleic acids, these platforms enable the efficient detection of diverse analytes, such as nucleic acids and small molecules. These advancements underscore the potential of NANs as foundational elements in advanced molecular sensing technologies.

NANs have demonstrated exceptional versatility and efficiency across diverse sensing platforms, including colorimetric, fluorescent, electrochemical, Raman, chemiluminescent, PEC, and photothermal sensors. These platforms exploit the high specificity of nucleic acid recognition and the catalytic activity of nanozymes to achieve sensitive, selective, and multifaceted detection of analytes such as small molecules, nucleic acids, proteins, microorganisms, and metal ions. Compared to other catalytic elements or detection methods, NANs-based sensing approaches offer unique advantages in multiple aspects (Table 2). Recent advancements highlight their applicability in precision diagnostics, environmental monitoring, and food safety. Future efforts will focus on enhancing multifunctionality, integrating portable and real-time detection systems, and improving performance in complex samples. These developments position NANs as pivotal tools in next-generation biosensing technologies.

## 5. Conclusions and Outlook

The integration of nucleic acid molecules has significantly enhanced the precision and sensitivity of nanozymes in biosensors. Nucleic acids, through their unique base-pair complementarity and higher-order structural formation, provide remarkable specificity in target recognition. The catalytic cleavage functions of DNAzymes and the binding capabilities of aptamers work synergistically with nanozyme materials to improve enzymatic activity and detection efficiency. As a result, NAN-based biosensors have excelled in molecular recognition, signal transduction, and detection performance, driving the rapid advancement of biosensor technologies.

Currently, nucleic acid-based sensors have demonstrated successful applications across various detection platforms, including colorimetric, fluorescence, electrochemical, Raman, and chemiluminescent systems. These sensors hold strong potential in fields such as environmental monitoring, food safety, and disease diagnosis, highlighting the broad application prospects of NANs.

However, challenges remain in further advancing the field of NANs, particularly the lack of a universal strategy for predicting the activity of nanomaterials. This has hindered the effective design and optimization of nanozymes, leading to a reliance on trial-and-error approaches that restrict progress. To overcome these challenges and propel the development of NAN biosensors, several future directions can be explored:

1. Development of Sensor Devices

Integrating NAN sensors into portable, flexible devices, such as paper-based and microfluidic technologies, will enable efficient POCT. Optimizing these designs for on-site and resource-limited scenarios can offer practical, real-world applications.

2. Functional Integration of Nucleic Acids and Nanozymes

Enhancing the functional integration of nucleic acids and nanozymes, such as through DNA-templated metallization or the discovery of novel nucleic acid recognition elements, will improve the performance and versatility of these sensors.

3. Stability Optimization of Nucleic Acids

While inorganic nanomaterials exhibit excellent chemical and thermal stability, nucleic acids are vulnerable to environmental factors in complex matrices like food, serum, or urine. Developing more stable nucleic acid components or incorporating stabilizers will be crucial for ensuring the reliability and robustness of NAN sensors in real-world applications.

4. Detection of Disease-Related Molecules

Expanding the scope of NAN research to include the detection of additional disease biomarkers, particularly in disease microenvironments, will open new avenues for in vivo sensing and imaging. This can significantly broaden the use of NANs in medical diagnostics and therapeutic monitoring.

5. Integration with Emerging Materials and Technologies

Incorporating novel nanomaterials and advanced technologies such as AI-assisted design and synthesis will introduce new strategies for developing highly sensitive, cost-effective NAN sensors. These innovations will extend detection ranges and improve sensor performance.

6. Control of Enzymatic Properties and Catalytic Efficiency

To expand the range of applications for NANs, an in-depth analysis of their enzymatic mechanisms and catalytic properties is essential. Improving the catalytic efficiency and specificity of nanozymes, including the exploration of new catalytic types (e.g., transferases-like, lyases-like, or isomerases-like), will enhance their versatility.

7. Biological Stability and Long-Term Performance

Despite significant interest in the medicinal value of NANs, long-term biological stability remains a major challenge. Overcoming this hurdle will be key to ensuring their widespread application in medical and environmental sensing.

8. Exploration of Cofactors of Nanozymes

Recent studies have highlighted the crucial role of cofactors in modulating the catalytic activity of nanozymes, including nucleic acids, polymers, and metal ions. These auxiliary factors significantly influence the morphology, stability, and enzymatic efficiency of nanozymes. Future research should focus on systematically investigating the diversity of cofactors, elucidating their regulatory mechanisms, and exploring rational design strategies to optimize their synergistic effects with nanozymes.

In summary, the innovative design and functional integration of NANs will continue to drive transformative progress in biosensor technology. By addressing the challenges of stability, catalytic efficiency, and device integration, NANs are poised to make revolutionary contributions to disease diagnosis, environmental monitoring, and life sciences research, solidifying their role as foundational tools in next-generation biosensing technologies.

## Figures and Tables

**Table 1:** A list of representative NANs.

Nanomaterials	Nucleic Acid Types	Mechanism	Influence	Ref.
MoS_2_ NSs	ssDNA (CEA aptamer, G20, T20, A20, C20)	The electron transfer process from TMB to H_2_O_2_ catalyzed by the MoS_2_ NSs was obviously accelerated via adding ssDNA.	Enhancement of POD-like activity	[31]
GO/Au	PBP2a aptamer	The phenomenon may be attributed to both aromatic stacking and electrostatic interaction with the substrate TMB.	Enhancement of POD-like activity	[32]
3DBC-C_3_N_4_	ssDNA (OTC aptamer, A22, T22, C22, and G22)	Facilitates TMB affinity for oxidation via electrostatic incorporation.	Enhancement of POD-like activity	[34]
CuNCs	DNA nanosheet	It is due to the content of intermediates generated after the introduction of nanozymes.	Enhancement of POD-like activity and specificity	[18]
Ti_3_C_2_	TBA aptamer	This is probably attributed to the π−π stacking between the benzene ring structure of OPD and the nucleobases of ssDNA.	Enhancement of POD-like activity and specificity	[35]
Porous Ti_3_C_2_	OA aptamer	Exposed Ti enhances DNA adsorption improves TMB affinity, and increases active intermediate •OH production.	Enhancement of POD-like activity and specificity	[36]
Fe_3_O_4_ NPs	DNA (Sm1, Sm2, Sm3)	Surface coverage was increased by bioconjugate DNA and Fe_3_O_4_ NP (physisorption < affinity coupling).	Enhancement of POD-like activity via DNA modification	[38]
Fe-cdDNA	ssDNA	The electrons are transferred through the DNA pathway that consists of H-bonds and through-space interactions (saturated bonds) to Fe(II).	The morphology and catalytic activity can be regulated by controlling synthesis conditions	[39]
Fe_3_O_4_ NPs/AuNPs	HCR products	HCR product with maximum negative phosphate charges exhibited highest binding for TMB oxidation.	Enhancement of POD-like activity	[41]
Fe_3_O_4_NP@pSiO_2_	hgDNA	The hgDNA enhances OPDA interaction with Fe_3_O_4_NP.	Enhancement of POD-like activity	[42]
AuNP	DNAzyme	Hydroxyl radicals generated from the reversible O-O bond cleavage of hydrogen peroxide on AuNPs oxidize adjacent DNA bases, converting them into radical cations. Upon contact of this charge with AR bound to the DNA, charge (hole) transfer occurs.	Enhancement of POD-like activity and specificity	[43]
CeO_2_	ssDNA	Phosphate-coated nanozyme enhance their interaction with TMB via electrostatic interactions.	DNA can both enhance and inhibit OXD-like of CeO_2_ activity depending on buffer and DNA concentration	[14,15]
CeO_2_	PCR products	The nucleic acids adsorb onto surfaces and induce aggregation of CeO_2_ NPs.	Reduces the OXD-like activity of CeO_2_ NPs	[50]
DAg/PtN DAu/PtN DCu/PtN DPtN	ssDNA (4 different nucleic acid sequences)	The four nanozymes are synthesized using DNA as a template.	Exhibits CAT-like activity	[58]
Au_25_ NCs	White adipocyte aptamers	Aptamers are mainly endowed with targeted and low-toxicity properties	It has SOD-like and CAT-like catalytic activity	[67]
Pt NPs	Oligonucleotides (A10, T10, C10, G10)	Pt^2+^ has coordination with nucleobase; the relative proportion of Pt^2+^ and Pt^0^ species determines enzyme activity.	Exhibits Laccase-like activity	[71]
C–Cu	Cytosine	The catalytic process may consist of the following four steps: 1. The substrates are adsorbed around it because of the large specific surface area of C–Cu. 2. The polyphenol substrates are oxidized and lost electrons with the reduction of Cu^2+^ to Cu^+^. 3. The active sites of C–Cu contacts and binds to O_2_, and electrons transfer to O_2_. 4. O_2_ gains electrons, combines with free protons in the reaction system, and is reduced to H_2_O with the oxidation of Cu^+^ to Cu^2+^, realizing the catalytic cycle of C–Cu.	Laccase-like activity can be controlled by synthesis conditions	[72]
Ag_2_O NPs	M17-F aptamer	Through interactions such as π-π stacking, hydrogen bonding, and other forces between nucleotide bases and the aromatic ring of the oxidized 2,4-DP substrate molecule, aptamers are able to adsorb increased amounts of the substrate and position it in close proximity to the cube-like Ag_2_O NPs.	All four base sequences enhance laccase-like activity	[74]
AuNPs	ssDNA/dsDNA	Nitrogenous bases in DNA can adsorb onto the surface of AuNPs. However, the higher surface charge density and rigidity of dsDNA make its binding to AuNPs more difficult compared to ssDNA.	ssDNA inhibits the GOx-like activity of AuNPs; dsDNA slightly perturbs the catalytic activity	[76]
AuNPs	G-rich DNA	Conformational changes in DNA nanomachines linked to the surface of AuNPs lead to changes in the exposed surface active area of the metal nanoparticles.	Reversible regulation of GOx-like activity	[78]

**Table 2:** Comparison of nucleic acid nanozymes with other catalytic and detection methods.

Comparison Criteria	Nucleic Acid Nanozymes (NANs)	Natural Enzymes	Artificial Nanozymes	Traditional Detection Methods (e.g., ELISA)
Catalytic Activity	High; tunable via sequence engineering	High, but susceptible to denaturation	High; stable	N/A ^1^
Specificity	High; target identification via nucleic acid hybridization or aptamer binding	Moderate; based on enzyme-substrate specificity	Moderate; depends on surface properties	High; dependent on antibody or sensor selectivity
Stability	High under diverse conditions	Low; sensitive to pH and temperature	Very high; robust in harsh conditions	Moderate; affected by environmental factors
Biocompatibility	High; based on nucleic acid-based systems	High, but potential immunogenicity	Moderate; concerns with metal toxicity	Moderate to high, depending on materials used
Cost and Scalability	Low cost; easy synthesis and modification	High cost; requires complex production	Moderate to high; depends on synthesis	Moderate to high; varies by detection system
Regulability and Flexibility	Highly programmable by sequence design	Limited; requires genetic engineering	Limited; depends on material properties	Moderate; relies on functionalization strategies
Detection Sensitivity	Very high; combinable with signal amplification	High, but requires optimized conditions	High; suitable for different detection methods	High; widely used in clinical diagnostics

^1^ N/A stands for Not Applicable.

## Data Availability

Data will be made available upon request.
